# Global prevalence of non-partner sexual violence against women

**DOI:** 10.2471/BLT.23.290420

**Published:** 2024-05-13

**Authors:** Lynnmarie Sardinha, Heidi Stöckl, Mathieu Maheu-Giroux, Sarah R Meyer, Claudia García-Moreno

**Affiliations:** a Department of Sexual and Reproductive Health and Research, World Health Organization, Avenue Appia 20, 1211 Geneva 27, Switzerland.; b Institute for Medical Information Processing, Biometry and Epidemiology, Faculty of Medicine, Ludwig-Maximilians-Universität München, Munich, Germany.; c Department of Epidemiology, Biostatistics and Occupational Health, School of Population and Global Health, McGill University, Montréal, Canada.

## Abstract

Sexual violence against women is a human rights violation and public health concern, with serious implications for women’s physical and mental health. Reducing non-partner sexual violence, including rape, sexual assault and other forms of non-contact sexual abuse, is one of the main indicators of the sustainable development goals. World Health Organization estimates, based on available prevalence data from 137 countries between 2000 and 2018, showed that, globally, 6% of women aged 15–49 years reported experiencing sexual violence in their lifetime from someone other than an intimate partner, with prevalence rates varying across regions. However, the reporting, measurement and documentation of the global extent of non-partner sexual violence against women is methodologically challenging, resulting in a gross underestimation of its magnitude and impact. To prevent and respond to this issue, policy-makers must consider interventions on education, access to relevant health-care services, public awareness, and effective and comprehensive legislation. To better estimate the prevalence of both sexual violence overall and non-partner sexual violence, it is essential to continue to strengthen the measurement of non-partner sexual violence, including the types of acts asked about and the mode of interviewing. Further research is needed to understand the cumulative impact of different forms of sexual violence on the lives of women and girls, including sexual violence during childhood and its associated risk with further exposure. Funding is required for more research and implementation of interventions to prevent and reduce all forms of violence against women and girls, including sexual violence.

## Résumé

Les violences sexuelles à l’égard des femmes constituent une violation des droits humains et un problème de santé publique qui ont de graves répercussions sur la santé physique et mentale des femmes. La réduction des violences sexuelles qui ne sont pas le fait d’un(e) partenaire, y compris le viol, l’agression sexuelle et d’autres formes d’abus sexuels sans contact, est l’un des principaux indicateurs des objectifs de développement durable. Selon les estimations de l’Organisation mondiale de la santé, qui se fonde sur les données de prévalence disponibles dans 137 pays entre 2000 et 2018, 6% des femmes âgées de 15 à 49 ans dans le monde ont déclaré avoir subi des violences sexuelles au cours de leur vie de la part d’une personne autre qu’un(e) partenaire intime, les taux de prévalence variant d’une région à l’autre. Toutefois, la déclaration, la mesure et la documentation de l’ampleur mondiale des violences sexuelles à l’égard des femmes qui n’impliquent pas de partenaire sont difficilement réalisables sur le plan méthodologique, ce qui entraîne une sous-estimation flagrante de leur ampleur et de leur impact. Pour prévenir et pallier ce problème, les décideurs politiques doivent envisager des interventions dans les domaines de l’éducation, de l’accès à des soins de santé appropriés, de la sensibilisation du public et de l’adoption d’une législation efficace et complète. Afin de mieux estimer la prévalence des violences sexuelles en général et des violences sexuelles qui ne sont pas le fait d’un(e) partenaire en particulier, il est essentiel de continuer à renforcer la mesure de ce dernier type de violences sexuelles, notamment en ce qui concerne les types d’actes sur lesquels portent les questions et le mode d’interrogation. Des recherches supplémentaires s’imposent pour comprendre l’impact cumulatif des différentes formes de violences sexuelles sur la vie des femmes et des filles, y compris les violences sexuelles subies pendant l’enfance et les risques associés à une exposition ultérieure. La recherche sur les interventions visant à empêcher et à réduire toutes les formes de violences à l’égard des femmes et des filles, y compris les violences sexuelles, et leur mise en œuvre doivent être davantage financées.

## Resumen

La violencia sexual contra las mujeres es una violación de los derechos humanos y un problema de salud pública, con graves consecuencias para la salud física y mental de las mujeres. Reducir la violencia sexual fuera de la pareja, incluidas las violaciones, las agresiones sexuales y otras formas de abuso sexual sin contacto, es uno de los principales indicadores de los Objetivos de Desarrollo Sostenible. Las estimaciones de la Organización Mundial de la Salud, basadas en los datos de prevalencia disponibles de 137 países entre 2000 y 2018, mostraron que, a nivel mundial, el 6% de las mujeres de 15 a 49 años informaron haber sufrido violencia sexual en su vida por parte de alguien que no era su pareja, con tasas de prevalencia que varían según las regiones. Sin embargo, la presentación de informes, la medición y la documentación del alcance mundial de la violencia sexual contra las mujeres fuera de la pareja es metodológicamente difícil, lo que resulta en una gran subestimación de su magnitud e impacto. Para prevenir y responder a este problema, los responsables de formular las políticas deben considerar la posibilidad de intervenir en la educación, el acceso a los servicios sanitarios pertinentes, la concienciación pública y una legislación eficaz y exhaustiva. Para estimar mejor la prevalencia tanto de la violencia sexual en general como de la violencia sexual fuera de la pareja, es esencial seguir reforzando la medición de esta última, incluidos los tipos de actos sobre los que se pregunta y el modo de entrevista. Se requiere más investigación para comprender el impacto acumulativo de las diferentes formas de violencia sexual en la vida de las mujeres y las niñas, incluida la violencia sexual durante la infancia y su riesgo asociado con una mayor exposición. Se requiere financiación para investigar más e implementar intervenciones que prevengan y reduzcan todas las formas de violencia contra mujeres y niñas, incluida la violencia sexual.

## ملخص

يعد العنف الجنسي ضد المرأة انتهاكًا لحقوق الإنسان، ومصدر قلق للصحة العامة، وتترتب عليه تبعات خطيرة على صحة المرأة الجسدية والعقلية. إن الحد من العنف الجنسي من غير الشركاء، بما في ذلك الاغتصاب والاعتداء الجنسي وغيره من أشكال الاستغلال الجنسي دون اتصال، يُعتبر أحد المؤشرات الرئيسية لأهداف التنمية المستدامة. أظهرت تقديرات منظمة الصحة العالمية، بناءً على البيانات المنتشرة المتاحة من 137 دولة بين عامي 2000 و2018، أن 6% من السيدات على مستوى العالم في الفئة العمرية من 15 إلى 49 عامًا، أبلغن عن تعرضهن للعنف الجنسي على مدى حياتهن من شخص آخر غير الشريك الحميم، مع معدلات انتشار تتنوع عبر المناطق. ورغم ذلك، فإن الكشف عن المدى العالمي للعنف الجنسي ضد المرأة من غير الشريك، فضلاً عن قياسه وتوثيقه، يمثل تحديًا منهجيًا، مما يؤدي إلى التقليل بشكل هائل من حجمه وتأثيره. وللحيلولة دون هذه المشكلة، والتعامل معها، يجب على واضعي السياسات أن يضعوا في اعتبارهم التدخلات المتعلقة بالتعليم، والحصول على خدمات الرعاية الصحية ذات الصلة، والوعي العام، والتشريعات الفعالة والشاملة. ولتقدير مدى انتشار العنف الجنسي بشكل عام، والعنف الجنسي من غير الشريك بشكل أفضل، من الضروري الاستمرار في دعم قياس العنف الجنسي من غير الشريك، بما في ذلك أنواع الأفعال التي يتم السؤال عنها وطبيعة إجراء المقابلات. هناك حاجة إلى مزيد من الأبحاث لفهم التأثير التراكمي للأشكال المختلفة للعنف الجنسي على حياة السيدات والفتيات، بما في ذلك العنف الجنسي أثناء الطفولة والمخاطر المرتبطة به مع المزيد من التعرض. يجب توفير التمويل لإجراء المزيد من الأبحاث عن التدخلات وتنفيذها لمنع وتقليل جميع أشكال العنف ضد السيدات والفتيات، بما في ذلك العنف الجنسي.

## 摘要

针对女性的性暴力是一种侵犯人权的行为，也是公共卫生问题，对女性的身心健康产生严重影响。减少非伴侣性暴力，包括强奸、性侵犯和其他形式的非接触性性虐待，是可持续发展目标的主要指标之一。世界卫生组织根据 2000 年至 2018 年之间 137 个国家可用的暴力行为相关数据进行了估计，结果表明，在全球范围内，15 至 49 岁女性中有 6% 在报告中称其在一生中遭受过来自亲密伴侣以外人员的性暴力，这一比例因地区而异。然而，报告、评估和记录全球范围内针对女性的非伴侣性暴力行为在方法上具有挑战性，导致严重低估了其规模和影响。为了预防和应对这一问题，政策制定者必须考虑在教育、获得相关卫生保健服务、提高公众意识以及制定有效且全面的法律方面采取干预措施。为了更好地估计所有性暴力行为和非伴侣性暴力行为的普遍存在情况，必须继续加强对非伴侣性暴力行为的评估，包括问及的行为类型和采访方式。需要进一步研究，以了解不同形式的性暴力行为对妇女和女孩生活的累积影响，包括童年时期遭遇的性暴力及与其进一步接触的相关风险。需要投入资金来开展更多研究并实施干预措施，来预防和减少针对妇女和女孩的一切形式的暴力行为（包括性暴力）。

## Резюме

Сексуальное насилие в отношении женщин – это нарушение прав человека и проблема общественного здравоохранения, которая влечет за собой серьезные последствия для физического и психического здоровья женщин. Снижение уровня сексуального насилия со стороны лиц, не являющихся партнерами, включая изнасилования, сексуальные посягательства и другие формы бесконтактного сексуального насилия, является одним из основных показателей достижения целей в области устойчивого развития. Согласно оценкам Всемирной организации здравоохранения, основанным на доступных данных о распространенности насилия в 137 странах за период с 2000 по 2018 год, во всем мире 6% женщин в возрасте 15–49 лет сообщили, что в течение жизни подвергались сексуальному насилию со стороны кого-либо, кроме сексуального партнера, причем показатели распространенности варьируются в зависимости от регионов. Однако составление отчетов, оценка и документирование глобальных масштабов сексуального насилия в отношении женщин со стороны лиц, не являющихся партнерами, сопряжены с методологическими трудностями, что приводит к серьезной недооценке его масштабов и последствий. Чтобы предотвратить и решить эту проблему, ответственные лица должны рассмотреть возможность принятия мер по просвещению, обеспечению доступа к соответствующим медицинским услугам, общественной осведомленности и принятию эффективного и всеобъемлющего законодательства. Для более точной оценки распространенности как сексуального насилия в целом, так и сексуального насилия со стороны лиц, не являющихся партнерами, необходимо продолжать укреплять систему измерения сексуального насилия со стороны лиц, не являющихся партнерами, включая определение типов совершаемых действий и способ проведения опроса. Для понимания совокупного воздействия различных форм сексуального насилия на жизнь женщин и девочек, включая сексуальное насилие в детстве и связанный с ним риск при дальнейшем воздействии, необходимо проведение дальнейших исследований. Для проведения дополнительных исследований и применения мер по предотвращению и сокращению всех форм насилия в отношении женщин и девочек, включая сексуальное насилие, необходимо финансирование.

## Introduction

Sexual violence against women is a human rights violation and public health concern worldwide.^1^^,^^2^ Such violence can occur in many circumstances and settings: within the home, in public spaces, educational institutions and workplaces, and can be exacerbated in conflict, displacement and other humanitarian contexts.^3^ Sexual violence takes many forms, including rape, attempted rape or sexual assault, conflict-related sexual violence, trafficking for the purposes of sex, and cyber sexual violence and other forms of non-contact sexual abuse.^1^ Although a substantial proportion of sexual violence experienced by women occurs within the context of marriage and other intimate relationships,^2^ non-partner sexual violence – that is, violence perpetrated by anyone other than an intimate partner, whether another family member, friend or acquaintance, neighbour or stranger – is also prevalent and has devastating and long-lasting effects on women’s health.

Sexual violence is associated with serious short- and long-term physical, sexual and reproductive, and mental health conditions, such as unwanted pregnancies, sexually transmitted infections, depression, post-traumatic stress disorder, suicide and other chronic health conditions.^4^^,^^5^ Further, sexual violence can be associated with the murder of women; for example, researchers studying femicides in South Africa estimated that 19.1% in 2009 and 8.7% in 2017 were sexual femicides.^6^ Sexual violence also has severe social and economic costs both for the individual and society, including the resulting lost productivity as well as costs to health, social, legal and other support services.^7^

Given the serious immediate and long-term impacts of sexual violence, preventing this violence from occurring but also ensuring access to adequate social, health, legal and other support services for affected women are urgent priorities. The 2030 United Nations sustainable development goal (SDG) target 5.2 calls on governments to “eliminate all forms of violence against all women and girls in public and private spheres,” with indicator 5.2.2 focusing on the prevalence of non-partner sexual violence.

In 2021, the World Health Organization (WHO) published a report on global, regional and national prevalence estimates that showed increased availability of data on both intimate partner violence and non-partner sexual violence against women.^8^ During 2000–2018, 137 countries completed at least one nationally or sub-nationally representative survey and/or study that measured non-partner sexual violence, compared with data available from less than 20 countries before 2000. The report demonstrated that, globally, an estimated 6% (95% uncertainty interval, UI: 4–9) or one in 17 women aged 15–49 years report having experienced non-partner sexual violence at least once in their lifetime since the age of 15 years (Table 1).^8^ Based on the world’s population in 2018,^9^ this finding equates to 160 million women (95% UI: 120–250 million) affected by the health, economic and social consequences of this violence. However, this is likely to be a gross underestimate.

**Table 1:** Global and regional prevalence of lifetime (from the age of 15 years) non-partner^a^ sexual violence against women aged 15–49 years

Sustainable development goal regions	Lifetime non-partner sexual violence point estimate, % (95% UI)	No. of countries and territories
**World**	6 (4–9)	137
**Sub-Saharan Africa**	6 (5–8)	33
**Northern Africa and Western Asia**		
Northern Africa	4 (2–9)	1
Western Asia	4 (2–9)	5
**Central and Southern Asia**		
Central Asia	2 (1–4)	3
Southern Asia	2 (1–3)	7
**Eastern and South-Eastern Asia**		
Eastern Asia	7 (2–21)	4
South-Eastern Asia	4 (2–8)	9
**Latin America and the Caribbean**	11 (7–16)	22
**Australia and New Zealand**	19 (9–36)	2
**Oceania (excluding Australia and New Zealand)**		
Melanesia	10 (5–22)	4
Federated States of Micronesia	12 (7–19)	5
Polynesia	12 (8–20)	3
**Europe and Northern America**		
Eastern Europe	6 (4–11)	9
Northern Europe	10 (6–16)	8
Southern Europe	7 (5–12)	13
Western Europe	8 (5–14)	7
Northern America	15 (5–40)	2
**Least developed countries**	5 (4–7)	35

UI: uncertainty interval.

^a^ Partner refers to any current or former husband or male intimate partner.

Source: World Health Organization.^8^

Despite the myriad, often life-long, consequences of sexual violence and its global prevalence, relatively little attention has been paid to measuring the magnitude and impact of non-partner sexual violence compared with sexual violence against women by intimate male partners. In this paper we focus on sexual violence by non-partners because it is a common experience in the lives of many women, contributes to their ill health in both the short and long term, and has received limited attention until recently from policy-makers.

## Data and measurement

The collection, analysis and reporting of robust data on the prevalence of non-partner sexual violence is the crucial first step to developing evidence-based prevention and response policies and programmes. The prevalence of intimate partner violence usually includes acts-based measures of sexual violence from partners, alongside physical and emotional violence. In contrast, most surveys that include items on non-partner sexual violence pose single general questions that relate to forced sex or forced sexual acts against a woman’s wishes, failing to adequately capture the different forms of non-partner sexual violence. For example, Demographic and Health Surveys (DHS; the main source of data on violence against women in low- and middle-income countries) only use a single question to ask women about their experience of sexual violence “as a child or as an adult,” capturing only the age of first “forced sexual intercourse”; only consider sexual violence resulting from physical force; and, until September 2019, only allowed the measurement of non-partner sexual violence if the first experience of “forced sexual intercourse or unwanted sexual act” was perpetrated by someone other than a (ex)husband or partner. Sexual violence by an intimate partner is measured more comprehensively and often with a different set of questions from non-partner sexual violence, even though women experience multiple forms of violence. Although the recording of specific acts is well established for the measurement of violence against women, including intimate partner violence, several surveys and studies continue to ask women if they have experienced sexual violence. Acts-based measures, that is, recording whether a specific act or behaviour (e.g. kicking, hitting with a fist, being physically forced to have sexual intercourse) has occurred, are preferred because of the enhanced disclosure and avoidance of a subjective interpretation of what constitutes violence. 

Other challenges affect the measurement of non-partner sexual violence. There is a lack of comparability between high-income, and low- and middle-income countries and regions. For example, although DHS data and data collected using the WHO Multicountry Study instrument^10^ or its adaptations are largely comparable between low- and lower-middle-income countries and regions, and the Violence against Women Survey^11^ conducted by the European Union Fundamental Rights Agency generates comparable data within Europe, data from these different surveys are not always comparable. This lack of standardization hampers the monitoring of progress towards reducing, and ultimately eliminating, sexual violence by 2030 as set out in the SDGs. An early review of the prevalence of non-partner sexual violence from 1983 to 2010 highlighted the wide variations in the definitions of non-partner sexual violence used in different studies and contexts.^1^ These definitions include case definitions of sexual violence, which give rise to different questions and often a focus on more severe forms of sexual violence, such as rape. Studies may also vary by the women sampled (all women regardless of their partnership status or only ever-partnered women), heterogenous age groups, recall period, list of perpetrators asked about, and inclusion or not of non-cohabiting boyfriends as partners. Together with the blame, shame, social stigma and multiple other challenges and repercussions that women face when disclosing sexual violence, the limitations of existing data and methods suggest that current figures grossly underestimate the true magnitude and impact of the problem.

There has been a slow but steady growth in the number of countries with population-based survey data on women’s experiences of sexual violence perpetrated by partners and non-partners; however, some regional gaps remain, for example in North Africa and the Middle East.^12^ There is also an encouraging growth in the recognition and use of internationally recommended ethical and safety guidelines by those producing data on the prevalence of non-partner sexual violence via violence-against-women surveys and modules. To support disclosure and adequately capture women’s experience of sexual violence, there is a need to provide comprehensive and specialized interviewer training, ensure privacy and confidentiality, prepare adequate referral systems and include acts-based questions in data collection.^2^

## Evidence synthesis to estimate prevalence 

The World Health Organization 2018 prevalence estimates^8^^,^^13^ used robust statistical methods that adjust for variations in survey design and methods of measuring non-partner sexual violence, presenting the first comparable global and regional lifetime prevalence estimates of non-partner sexual violence within the SDG reporting period. These statistical methods involved two steps (details published elsewhere).^8^^,^^12^

First, WHO conducted a comprehensive systematic review of all available global prevalence data, including published literature in four medical and social science databases, and the websites and metadata repositories of national statistics offices and large-scale surveys. WHO analysed national data from 75 DHS and two Reproductive Health Surveys, as well as the WHO Multicountry Study^10^ and other publicly available microdata and data sets from national statistical repositories. All data on (non-partner) sexual violence from population-based studies, representative at national or subnational level and conducted between 2000 and 2018, were eligible for inclusion and extracted to the WHO Global Database on the Prevalence of Violence against Women.^14^

Second, WHO developed a Bayesian multilevel model to estimate lifetime non-partner sexual partner violence from the age of 15 years by age and country, for all 21 Global Burden of Disease regions.^15^ This framework accounts for heterogeneous age groups, adjusts for differences in outcome definition, and weights survey data depending on its representativeness and/or geographical strata.

Data from 227 studies encompassing responses from around 1.3 million girls and women (aged 15–49 years) across 137 countries and areas, representing 88% of the global population of women, informed the lifetime prevalence estimates for non-partner sexual violence from the age of 15 years. Results highlight considerable regional variation in the prevalence of non-partner sexual violence (Table 1).^8^ The large difference in non-partner sexual violence prevalence between higher-income countries and regions such as Australia, New Zealand and Northern America and the rest of the world need to be interpreted cautiously, taking several factors into account. Although these estimates may reflect an actual difference in prevalence, they also potentially result from major gaps in the available data, variations in measurement and reporting approaches (including single questions focused only on rape and/or attempted rape versus multiple questions based on more comprehensive definitions), interviewer training, survey type (multipurpose versus those focused on violence against women) and, importantly, social and cultural norms that may support or supress women’s disclosure of sexual violence.

Although statistical methods as described here can enable more comparable estimates of prevalence, fundamental improvements in survey measures of non-partner sexual violence are vital if we are to understand the scope of the problem and identify women who are most affected. This is key to the development of targeted, effective and sustainable interventions. This includes the need for a clear definition and conceptualization of non-partner sexual violence that goes beyond measuring rape, captures different forms of coercion (not just physical force), and includes attempted rape and other forms of contact and non-contact sexual violence that are delineated from sexual harassment. Because women experience multiple and overlapping forms of violence from partners and non-partners, a measure that captures sexual violence by intimate partners and any other perpetrators in a comparable way would be useful. Such an acts-based measurement tool must be internationally standardized, as is the case for intimate partner violence, to allow comparable estimates between countries and regions and to monitor trends.

## Policy and research implications

Despite robust evidence that intimate partner violence can be prevented,^16^ only a few programmes focused on sexual violence prevention have been developed and rigorously evaluated, and these predominantly occur in high-income countries.^17^

Discriminatory gender norms and institutions that foster and perpetuate violence against women are key driving factors for the occurrence and lack of reporting of non-partner sexual violence.^18^ Women who report rape and sexual assault are often blamed for being in the wrong place at the wrong time, wearing the wrong clothes or not fighting back against the assault.^19^ In addition, the strong taboos and social stigma around sexual violence contribute to shame and self-blame, and discourage women from disclosing their experiences even to their family and friends, or from seeking formal help from the legal or health systems. 

Efforts to prevent sexual violence from occurring are paramount. Primary prevention of sexual violence includes early intervention with adolescents through a whole-school approach that challenges gender-inequitable attitudes and practices in all school processes and formal curricula. Elements of effective school-based prevention programmes should include: ensuring that schools are accessible, safe and zero-tolerance for violence environments for all young people; teaching healthy and equitable relationships; covering issues around self-esteem, consent and safe sex; and recognizing signs of violence/abuse and coercion.^20^ Community-based interventions sustained over time could also change and form gender-equitable attitudes among men and women towards a more healthy norm,^21^ especially when combined with multicomponent interventions.^22^ Because gender perceptions and attitudes are formed early in life, and child sexual abuse and other forms of child maltreatment and child neglect are often predictors for later perpetration of sexual violence, parenting and other programmes to reduce child abuse and neglect are also relevant in the prevention or reduction of non-partner sexual violence.^22^

Given the substantial physical and mental health impacts as well as high prevalence of non-partner sexual violence, health services have an important role to play in identifying the problem and in providing immediate care (including emergency contraception and prophylaxis for human immunodeficiency virus infection and other sexually transmitted infections in case of rape), psychological support and referrals to specialized support services.^2^ Health-care providers can access guidance that includes, but is not limited to, comprehensive post-rape care and services including mental health care through survivor-centred approaches.^23^^,^^24^ A multisectoral referral pathway to other support services also needs to be developed and strengthened, and these services should always be available and accessible.^25^ Greater availability and training of sexual assault nurse examiners (or other specially trained health personnel) that can provide survivor-centred, comprehensive care to sexual assault survivors, as well as access to 24/7 sexual assault care centres with specialized, multidisciplinary and longitudinal care for survivors, need to be supported.

Progress has been made in terms of the recognition and awareness of sexual violence and its harmful effect on women and the overall society by the general public; however, policy has been slow to change. Social media movements such as Me Too (#metoo) and *Ni Una Más* (#niunamas: Not One More) have brought attention to the many forms of sexual violence that women and girls experience, although accountability of perpetrators remains elusive. Although there has been an increase in the number of countries (155 in May 2024) with laws and policies aimed at responding to and preventing violence against women, the quality and comprehensiveness of legislation with regards to definitions of sexual violence, the effective implementation of any legislation, the evidence required for prosecution and subsequent penalties, and the availability of support services for women all remain inadequate in a large number of countries.^18^ Sexual violence is one of the most poorly prosecuted crimes, even in high-income countries with well-resourced judicial systems. There is an urgent requirement for police, legal and judicial systems to be more responsive to the needs of sexual violence survivors, and for the implementation of laws that promote and support gender equality and address violence against women.

Adequate and stable funding to women’s organizations and movements that have been at the forefront of providing services, and advocating better and stronger legislation, is needed.^18^ For example, a systematic review found that funding associated with the 1994 United States Violence Against Women Act was key in reducing the prevalence of non-partner sexual violence in the United States of America.^22^

## Conclusion

WHO has been working with its partners to strengthen the measurement of violence against women and girls, and will also be producing global, regional and national prevalence estimates of non-partner sexual violence in 2025. Further research is needed to understand the cumulative impact of different forms of sexual violence on the lives of women and girls, including sexual violence during childhood and its associated risk with further exposure. Funding is required for more research on and implementation of interventions to prevent and reduce all forms of violence against women and girls, including sexual violence. 
